# Delineating the genetic landscape of Charcot–Marie–tooth disease in Türkiye: Distinct distribution, rare phenotypes, and novel variants

**DOI:** 10.1111/ene.16572

**Published:** 2025-01-08

**Authors:** Arman Cakar, Ayse Candayan, Gulandam Bagırova, Zehra Oya Uyguner, Serdar Ceylaner, Hacer Durmus, Esra Battaloglu, Yesim Parman

**Affiliations:** ^1^ Neuromuscular Unit, Neurology Department, Istanbul Faculty of Medicine Istanbul University Istanbul Turkey; ^2^ Department of Molecular Biology and Genetics Bogazici University Istanbul Turkey; ^3^ Molecular Neurogenomics Group VIB Center for Molecular Neurology, VIB Antwerp Belgium; ^4^ Department of Biomedical Sciences University of Antwerp Antwerp Belgium; ^5^ Department of Medical Genetics, Istanbul Faculty of Medicine Istanbul University Istanbul Turkey; ^6^ Institute of Health Sciences Istanbul University Istanbul Turkey; ^7^ Medical Genetics Intergen Genetics Laboratory Ankara Turkey

**Keywords:** CMT, genomics, hereditary, polyneuropathy, population genetics, sequencing, Turkey

## Abstract

**Background:**

Charcot–Marie‐Tooth (CMT) disease is the most common inherited neuropathy. In this study, we aimed to analyze the genetic spectrum and describe phenotypic features in a large cohort from Türkiye.

**Methods:**

Demographic and clinical findings were recorded. Patients were initially screened for *PMP22* duplication. Targeted sequencing or whole‐exome sequencing was performed in duplication‐negative patients.

**Results:**

Overall, 311 patients from 265 families were included. Demyelinating CMT (67.4%) was more common than axonal (20.5%) and intermediate subtypes (11.7%). *PMP22* duplication was the most frequent mutation, followed by pathogenic variants in *GJB1*, *MFN2*, *SH3TC2*, and *GDAP1* genes. *MPZ*‐neuropathy was rare in our cohort (3.0%). Interestingly, CMT4 is the second most common type after CMT1. Lower extremity weakness and foot deformities were the most frequent presenting complaints. Striking clinical features included a high frequency of scoliosis in *SH3TC2*, peripheral hyperexcitability in *HINT1*, and central nervous system findings in *GJB1*. Autosomal recessive CMT subtypes had higher CMTESv2 scores when compared to autosomal dominant ones (12.39 ± 4.81 vs. 8.36 ± 4.15, *p*: 0.023). Twenty‐one patients used wheelchairs during their last examination. Among them, 16 had an autosomal recessive subtype. Causative variants were identified in 31 genes, including 28 novel pathogenic or likely pathogenic changes.

**Conclusions:**

Our findings provided robust data regarding the genetic distribution of CMT in Türkiye, which may pave the path for building population‐specific diagnostic gene panels. Rare autosomal recessive subtypes were relatively frequent in our cohort. By analyzing genotype–phenotype correlations, our data may provide clinical clues for clinicians.

## Graphical abstract

This study provided robust data regarding the genetic distribution of CMT in Türkiye, which is essential to create population‐specific diagnostic workflows to increase the diagnosis rate. Rare autosomal recessive subtypes were relatively frequent in the cohort. By analyzing clinical findings and genotype–phenotype correlations, particularly in autosomal recessive subtypes with complex clinical features, our data may provide diagnostic clues for clinicians.

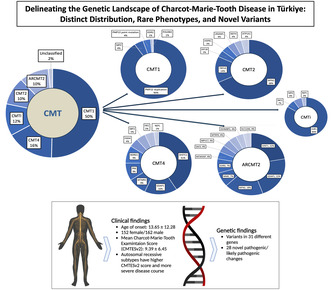

## INTRODUCTION

Charcot–Marie‐Tooth (CMT) disease is the most common inherited neuropathy with prevalence of 17.69 in 100,000 individuals [1]. The classification of CMT is based on nerve conduction study findings (NCS) and inheritance pattern. Demyelinating CMT types, CMT1 (autosomal dominant) and CMT4 (autosomal recessive), stand for patients with a median motor nerve conduction velocity (NCV) less than 38 m/s, and axonal CMT forms, CMT2 (autosomal dominant) and AR‐CMT2 (autosomal recessive) are defined by a median motor NCV higher than 38 m/s. Furthermore, Intermediate CMT (CMTi) is defined by NCV between 25 and 45 m/s [2]. However significant overlap can be observed between other hereditary neuropathies [3].

Clinically, most cases with CMT manifest with a slowly progressive symmetric distal weakness and atrophy in the lower limbs accompanied by skeletal deformities such as *pes cavus* and hammer toes, usually beginning in the first to third decade. Sensory symptoms are rare; however, they may be observed in the neurological examination [2]. Another presentation of CMT, which is more frequent with recessive forms, is with an earlier onset, delayed motor milestones, and progressive walking difficulties resulting in loss of ambulation. Additional features, such as central nervous system involvement, vocal cord paralysis, deafness, kyphoscoliosis, and optic atrophy may be present depending on the subtype [4, 5].

Owing to the progress in molecular genetics, such as next‐generation sequencing (NGS), more than 140 disease‐causing genes have been identified so far [6]. On the contrary, around 90% of all genetically diagnosed CMT cases have pathogenic variants in four genes: *PMP22*, *MFN2*, *MPZ*, and *GJB1* [7]. Therefore, sequential screening of the most frequent genes depending on the inheritance pattern and electrophysiological features was initially used for genetic diagnosis prior to the NGS era. Although this method is cost‐effective, it can be highly time‐consuming. Therefore, a common diagnostic strategy is analyzing a virtual gene panel of disease‐causing genes in whole‐exome sequencing (WES) or whole‐genome sequencing (WGS) data after excluding *PMP22* duplication with Multiplex Ligation‐dependent Probe Amplification (MLPA) [8]. Notably, even with extensive analyses with commercially available genomic techniques, only around 60% of CMT cases receive a genetic diagnosis [9].

CMT is mostly inherited in an autosomal dominant pattern. In countries with a high rate of consanguineous marriages, like Türkiye, autosomal recessive forms are also frequent. This study aimed to highlight the distribution of genetically diagnosed patients and describe the phenotypic spectrum in a large cohort.

## METHOD

### Clinical evaluation and statistical analysis

Our study includes patients who were followed at the Neuromuscular Unit of Istanbul University between 1995 and 2024. The clinical dataset included demographic features, medical history including age at disease onset, first symptom, wheelchair dependency, age at ambulation loss, neurological examination, and median motor nerve conduction velocity findings. Regarding neurological examination, distal weakness, atrophy, reduced deep tendon reflexes, and mild sensory signs were considered as typical features. The remaining clinical findings, such as cranial nerve involvement, severe sensory ataxia, and central nervous system signs were regarded as atypical findings and recorded separately. CMT examination score version 2 (CMTESv2) was used to measure disease severity in 289 patients. Patients clinically diagnosed with hereditary liability to pressure palsies or distal hereditary motor neuropathy (dHMN) were excluded from the CMT subtype distribution, even if they harbor a pathogenic variant in the CMT‐associated genes. Furthermore, patients exhibiting CMT phenotype with variants in genes typically not associated with isolated CMT were excluded from the analyses and discussed separately. SPSS version 26 was used for statistical analysis. Data distribution was assessed for normality using the Kolmogorov–Smirnov and Shapiro–Wilk tests and analyzing skewness, and kurtosis. Comparison of means between two independent groups was performed with a Student's *t*‐test, or Mann–Whitney *U*‐test when appropriate.

### Genetic tests

Initially, all patients with a clinical diagnosis of CMT were screened for *PMP22* duplication either using short tandem repeat (STR) markers or MLPA (P033‐CMT1). In a number of patients, additional CMT genes that are relatively common in the Turkish population (*MPZ*, *GJB1*, *MFN2*, and *GDAP1*) were sequentially screened based on their neuropathy type and inheritance pattern using Sanger sequencing. The patients with negative results were subjected to WES or targeted gene panel sequencing. Collaborating research or diagnostic laboratories provided genetic findings, which had been analyzed using WES or in‐house gene panels. Variant calling was done by each research laboratory separately; protocols, consumables, and pipelines differ between these institutions. Detailed protocols can be provided upon request. Recurrent pathogenic variants identified in patients were verified in index cases and available family members using Sanger sequencing. American College of Medical Genetics (ACMG) criteria were used to classify the pathogenicity of novel alterations [10]. The study was approved by the Istanbul Medical Faculty Clinical Research Ethics Committee (Approval number: 2019/770) and complies with the agreements of the Declaration of Helsinki. Patients provided written informed consent before the procedures.

## RESULTS

### Frequency of CMT subtypes and overview of the cohort

**FIGURE 1:**
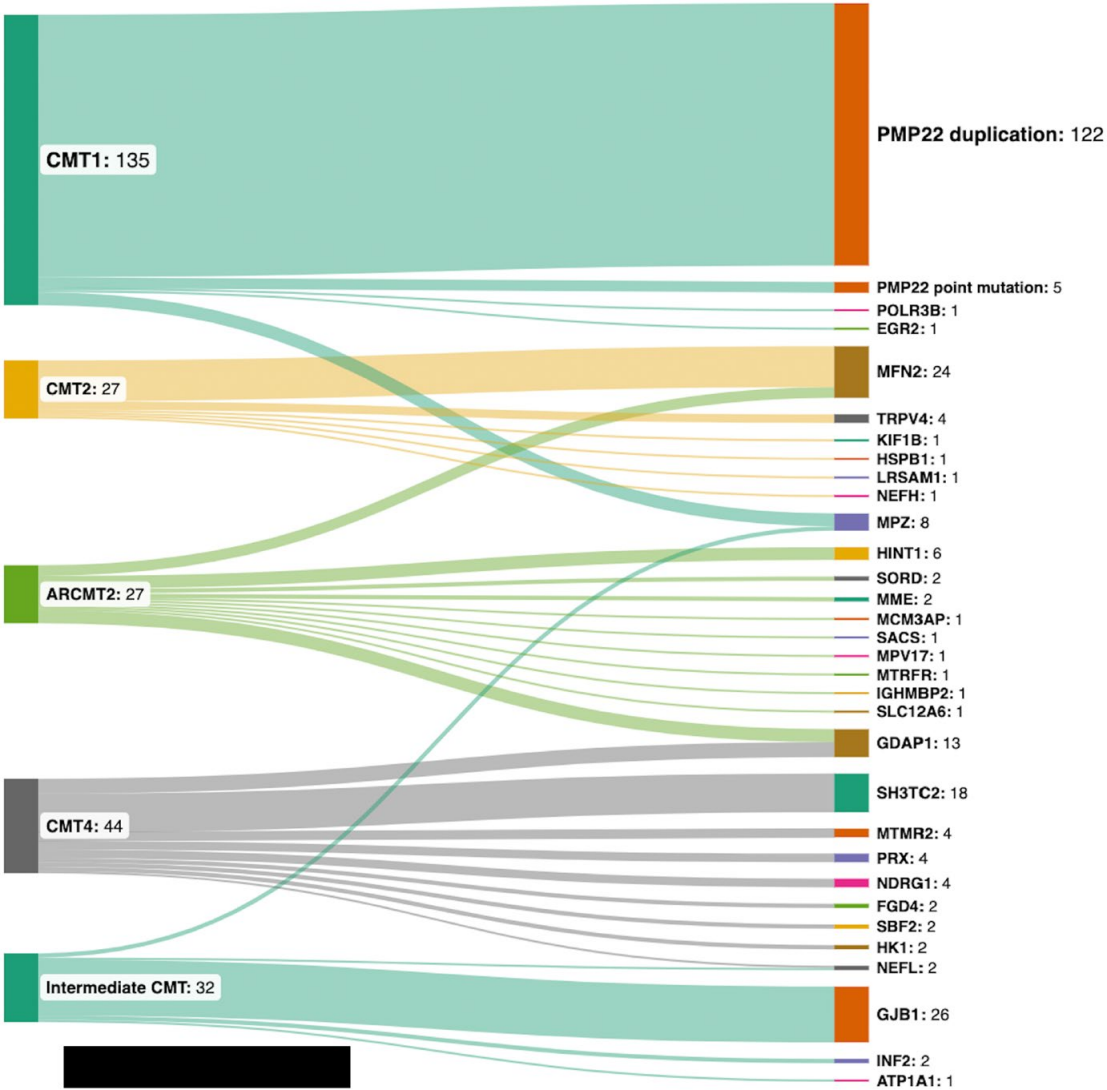
Overview of the study cohort and subtypes. Numbers show identified families.

Overall, 315 patients from 269 families with a clinical diagnosis of CMT and harboring variants in 31 different genes were included in this study. Of note, four patients with variants in genes typically not associated with isolated CMT were excluded from the further analyses. We have identified 28 novel variants that are classified as pathogenic or likely pathogenic variants according to the ACMG criteria (Table S1). The most frequent subtype was CMT1 (135 families), followed by CMT4 (44 families), CMTi (32 families), CMT2 (27 families), and AR‐CMT2 (27 families). We further identified 12 more families with variants in CMT‐related genes such as *HINT1* (six families), *SORD* (five families), and *TRPV4* (one family) by screening patients with a dHMN phenotype, which were excluded from this study. The most commonly associated genes were *PMP22*, *GJB1, MFN2*, *SH3TC2*, and *GDAP1*, respectively. Interestingly, only eight families were identified with *MPZ* variants (3%). The frequency of CMT subtypes is summarized in Figure 1. Duplication of *PMP22* accounted for 90% (122/135) of all CMT1 families. Among CMT2, the most frequent subtype was CMT2A2A, caused by monoallelic variants in *MFN2* (70%). The distribution of subtypes in recessive CMT forms was more heterogeneous. Among 70 families with either AR‐CMT2 or CMT4, variants were identified in 20 different genes.

The mean age of onset was 13.65 ± 12.28 years (range 1–57), and 149 out of 314 patients were female. Patients with autosomal recessive CMT forms have an early disease onset (mean: 6.39 ± 5.98, range 1–29 years). Symptoms related to lower limb weakness or skeletal deformities (230 patients) were the most common presenting complaint, followed by delayed motor milestones (38 patients). Among patients with delayed motor milestones, 24 had an autosomal recessive CMT subtype. Parents were consanguineous in 108 patients. The mean age of patients at the time of neurological examination was 32.85 ± 16.54 years (range 3–85). The most common skeletal deformity was *pes cavus* (251 patients), followed by hammer foes (130 patients). Twenty‐one patients were using wheelchair during the last visit.

### Frequent CMT subtypes

#### PMP22

Among 135 patients with *PMP22* duplication (CMT1A), 90 had disease onset in the first or second decade, and all, except for one, were ambulatory. Partial duplications of the *PMP22* gene were causative in seven patients from five families. The clinical features of these patients were indistinguishable from those with the conventional 1.5 Mb duplication on chromosome 17p11.2. Five patients had four different heterozygous missense or protein‐truncating variants in *PMP22* (CMT1E) (Table S1). The age of disease onset was in the first decade in these patients, and severe sensory ataxia dominated the phenotype in three. Interestingly, two patients with heterozygous p.(Gly94Alafs*17) and p.(Ser72Leu) variants had ophthalmoparesis.

#### GJB1

Among 32 patients with a pathogenic variant in the *GJB1* gene, 10 were female. Females had a later disease onset compared to males (25.11 ± 14.37 vs. 15.44 ± 10.96, *p*: 0.048). One male patient with a hemizygous p.(Tyr211His) variant had a history of stroke‐like episodes with white matter lesions in cranial MRI, two patients had mild intellectual disability (one male with a hemizygous p.Arg15Gln and a female with a heterozygous p.(Arg230Cys) variant) and one female with a novel heterozygous p.(Lys260Glufs*16) variant had brisk tendon reflexes also suggesting central nervous system (CNS) involvement. In a mean disease duration of 20.31 ± 14.56 years, all patients remained ambulatory. The mean median motor NCV was in the intermediate range both among males and females (37.17 ± 8.84 vs. 33.67 ± 6.29, *p*: 0.41). CMTESv2 scores were significantly higher in males (9.41 ± 3.48 vs. 5.11 ± 2.71, *p*: 0.010) (Table 1).

**TABLE 1:** Clinical and genetic features of the patients harboring variants in the most frequently associated genes.

Causative gene	*PMP22*	*GJB1*		*MFN2*	*SH3TC2*	*GDAP1*	*MPZ*
		Male	Female				
Subtypes (number of patients/families)	CMT1A (135/122) CMT1E (5/5)	CMTX1 (32/26)		CMT2A2A: (30/19) CMT2A2B: (5/5)	CMT4C (22/18)	CMT4A (7/7) ARCMT2K (11/6)	CMT1B (6/6) CMTDID (2/2)
Age at onset (mean ± STDEV)	CMT1A: 18.05 ± 13.43 CMT1E: 4.50 ± 2.78	14.95 ± 10.97	25.20 ± 13.55	CMT2A2A: 8.20 ± 5.81 CMT2A2B: 5.40 ± 5.18	8.27 ± 5.83	CMT4A: 5.30 ± 3.44 ARCMT2K: 3.21 ± 2.04	CMT1B: 14.83 ± 36.04 CMTDID: 31.00 ± 43.43
Age at examination (mean ± STDEV)	CMT1A: 36.86 ± 16.55 CMT1E: 25.60 ± 14.19	38.91 ± 14.45	34.70 ± 14.55	CMT2A2A: 26.33 ± 18.05 CMT2A2B: 11.20 ± 4.19	26.36 ± 14.20	CMT4A: 19.14 11.36 ARCMT2K: 29.09 ± 16.22	CMT1B: 39.83 ± 18.95 CMTDID: 39.50 ± 20.51
Sex (female/male)	CMT1A: 77/58, CMT1E: 1/4	10/22		CMT2A2A: 20/10, CMT2A2B: 3/2	15/7	CMT4A: 3/4, ARCMT2K: 3/8	CMT1B: 3/3, CMTDID: 0/2
Parental consanguinity (number of patients)	CMT1A: 17, CMT1E: 1	3		CMT2A2A: 5, CMT2A2B: 5	17	CMT4A: 4, ARCMT2K: 11	CMT1B: 1, CMTDID: 1
DMM (number of patients)	CMT1A: 0, CMT1E: 1	0	0	CMT2A2A: 5, CMT2A2B: 2	3	CMT4A: 3, ARCMT2K: 1	CMT1B: 1, CMTDID: 0
Pes cavus (number of patients)	CMT1A: 119, CMT1E: 3	20	9	CMT2A2A: 19, CMT2A2B: 4	19	CMT4A: 4, ARCMT2K: 8	CMT1B: 4, CMTDID: 2
Hammer toes (number of patients)	CMT1A: 58, CMT1E: 1	11	5	CMT2A2A: 10, CMT2A2B: 2	10	CMT4A: 1, ARCMT2K: 5	CMT1B: 4, CMTDID: 1
Scoliosis (number of patients)	CMT1A: 8, CMT1E:1	2	0	CMT2A2A: 1, CMT2A2B: 0	17	CMT4A: 3, ARCMT2K: 1	CMT1B: 2, CMTDID: 0
Other foot deformities (number of patients)	CMT1A: 2, CMT1E: 0	0	0	CMT2A2A: 5, CMT2A2B: 1	1	CMT4A: 0, ARCMT2K: 2	CMT1B: 0, CMTDID: 0
Tremor (number of patients)	CMT1A: 8, CMT1E:1	6	3	CMT2A2A: 5, CMT2A2B: 1	1	CMT4A: 0, ARCMT2K: 3	CMT1B: 2, CMTDID: 0
Ambulation loss (number of patients)	CMT1A: 1, CMT1E: 0	0	0	CMT2A2A: 3, CMT2A2B: 1	3	CMT4A: 3, ARCMT2K: 1	CMT1B: 1, CMTDID: 0
Cranial nerve involvement (number of patients)	CMT1A: NA CMT1E: Ophthalmoparesis (2)	NA	NA	CMT2A2A: Optic atrophy (2), vocal cord paralysis (2), ophthalmoparesis (1) CMT2A2B: Optic atrophy (1), vocal cord paralysis (1)	Vocal cord paralysis, facial paralysis	CMT4A: Vocal cord paralysis (3) ARCMT2K: Sensorineural hearing loss (1)	NA
CMTESv2 (mean ± STDEV)	CMT1A: 7.49 ± 3.39 CMT1E: 13.00 ± 4.74	9.47 ± 3.60	5.50 ± 2.84	CMT2A2A: 10.37 ± 4.35 CMT2A2B: 12.00 ± 8.00	11.90 ± 4.49	CMT4A: 14.00 ± 2.83 CMT2K: 13.00 ± 4.56	CMT1B: 11.17 ± 2.93 CMTDID: 7.00 ± 0
Median motor NCV (mean ± STDEV)	CMT1A: 21.10 ± 7.78 CMT1E:1: 12.14 ± 14.28	37.17 ± 8.84	33.67 ± 6.29	CMT2A2A: 48.64 ± 6.28 CMT2A2B: 53.40 ± 3.00	21.95 ± 9.22	CMT4A: 22 m/s (inexcitable in 6 patients) ARCMT2K: 50.57 ± 5.93	CMT1B: 14.55 ± 7.46 CMTDID: 37.05 ± 0.07
Other clinical features	NA	ID, multiple sclerosis‐like clinical features, pyramidal signs, small fiber neuropathy		CMT2A2A: ID, pyramidal signs CMT2A2B: Multiple symmetric lipomatosis	NA	ARCMT2K: Pyramidal signs	CMT1B: Conduction blocks in nerve conduction studies
Variant types (Number of families)	CMT1A: Duplication (117), partial duplication (5) CMT1E: TV (2), Missense (1)	Missense (18) TV (3) 5′ UTR (2)		CMT2A2A: Missense (13) CMT2A2B: Missense (5)	TV (7) Missense (4) Splice donor (1)	CMT4A: TV (4) ARCMT2K: Missense (4)	CMT1B: Missense CMTDID: Missense
Zygosity (Families)	CMT1A: Heterozygous (122) CMT1E: Heterozygous (5)	Hemizygous (22, males)	Heterozygous (10, females)	CMT2A2A: Heterozygous (19) CMT2A2B: Homozygous (4), compound heterozygous (1)	Homozygous (16) Compound heterozygous (2)	CMT4A: Homozygous (7) ARCMT2K: Homozygous (6)	CMT1B: Heterozygous (6) CMTDID: Heterozygous (2)

Abbreviations: CMT, Charcot‐Marie‐Tooth; CMTESv2, Charcot‐Marie‐Tooth examination score Version 2; DMM, Delayed motor milestones; F, female; ID, Intellectual disability; M, Male; NA, Not applicable; NCV: Nerve conduction velocity; STDEV, Standard deviation; TV, Truncating variant.

#### MFN2

Thirty‐five patients had pathogenic variants in the *MFN2* gene. The age of disease onset was in the first or second decade in all patients except for three. Four patients became wheelchair‐dependent during the second decade. Four patients, two with a heterozygous p.(Arg364Trp), one with a heterozygous p.(Arg94Trp), and one with a novel homozygous p.Val91Leu variant, had optic atrophy. Vocal cord involvement was observed in three patients. Eighteen families had monoallelic, and five had biallelic variants in the *MFN2* gene. Two patients with a homozygous p.(Arg707Trp) variant, had an identical phenotype of multiple symmetric lipomatosis with mild sensory‐predominant neuropathy (Table 1).

#### SH3TC2

Among 22 patients with pathogenic variants in the *SH3TC2* gene, only one had a disease onset later than 20 years. Scoliosis or kyphoscoliosis were present in 17 patients. Three patients lost ambulation in a mean disease duration of 18.09 ± 15.06 years. We identified five different missense and nine truncating variants either in a homozygous or compound heterozygous state (Table S1).

#### GDAP1

Eighteen patients had pathogenic variants in the *GDAP1* gene. The disease onset was in the first decade in all except for one. The severity of the disease was striking in terms of cranial nerve involvement (four patients) and high frequency of ambulation loss (seven patients) (Table 1). Median motor NCV was in the axonal range in 11 patients and demyelinating in seven. We identified two were novel (p.[Phe289Leufs*5] and p.[Phe289Ser]). Three families carrying the homozygous founder p.(Phe263Leufs*22) variant exhibited demyelinating neuropathy, while three others with a homozygous p.Asp149Tyr variant had axonal neuropathy.

#### MPZ

Eight patients had pathogenic variants in the *MPZ* gene. Disease onset was in adulthood (>20 years) in four patients. On the contrary, two with heterozygous p.[Thr34Ile] and compound heterozygous of p.[Val42del] and p.[Ala221Thr] variants had an infantile‐onset (<3 years). The mean median motor NCV of the adult‐onset cases was 26.52 ± 12.18, although two had relatively lower values (15 and 17 m/s) (Table 1). All patients were heterozygous for the *MPZ* variants except for one carrying both p.(Val42del) and p.(Ala221Thr) variants in compound heterozygosity. One patient had nerve conduction blocks in NCS and was misdiagnosed as chronic inflammatory demyelinating polyneuropathy (CIDP).

### Other subtypes

Only two CMT1 patients had variants in genes other than *PMP22* and *MPZ*. One patient had a pathogenic variant in *EGR2* (heterozygous p.[Arg381His]) and the other in the *POLR3B gene* (heterozygous p.[Arg1046His]). The patient with the pathogenic *EGR2* variant had an early disease onset with motor delay and unobtainable responses in NCS. The patient with the *POLR3B* variant also presented in the first decade of life with walking problems and was further diagnosed with hypogonadotropic hypogonadism.

Apart from the frequent *SH3TC2* and *GDAP1* subtypes, we identified 21 patients from 19 families with CMT4 harboring biallelic variants in seven genes. All patients presented in the first decade of life except for two harboring variants in *HK1* and *FDG4* genes (Table 2). Patients with pathogenic *PRX* variants showed prominent sensory ataxia. Three out of four probands had the same homozygous p.(Arg1070*) variant in the *PRX* gene. Four probands had homozygous pathogenic variants in *NDRG1*, and three with a Romani origin had the recurrent p.(Arg148*) variant. Interestingly, two out of three patients with pathogenic variants in the *FGD4* gene exhibited ptosis without ophthalmoplegia and one had tongue fasciculations.

**TABLE 2:** Clinical and genetic features of the patients with demyelinating autosomal dominant (CMT1) and autosomal recessive (CMT4) Charcot‐Marie‐Tooth subtypes.

Causative gene	*MTMR2*	*PRX*	*NDRG1*	*FGD4*	*SBF2*	*HK1*	*NEFL*	*POLR3B*	*EGR2*
Number of patients/families	5/4	4/4	4/4	3/2	2/2	2/2	1/1	1/1	1/1
Age at onset (years median/range)	1.5/between 1 and 4	2/between 1 and 4	4/between 2 and 7	2/between 2 and 11	2 and 3	5 and 13	7	3	2
Age at examination (years median/range)	25/between 7 and 38	16/between 8 and 31	13/between 13 and 23	20/between 14 and 32	16 and 25	18 and 20	33	16	6
Sex (female/male)	3/2	1/3	2/2	2/1	1/1	0/2	Male	Female	Male
Parental consanguinity (number of patients)	4	4	3	3	2	2	Yes	Yes	No
Positive family history (number of patients)	3	3	2	2	0	2	Yes	No	No
DMM (number of patients)	4	3	1	2	1	0	No	Yes	Yes
Pes cavus (number of patients)	3	2	3	3	1	2	Yes	Yes	No
Hammer toes (number of patients)	1	0	0	3	1	2	Yes	No	No
Scoliosis (number of patients)	1	2	1	0	1	0	Yes	No	No
Other foot deformities (number of patients)	2	1	1	0	1	0	No	No	No
Tremor (number of patients)	0	0	0	0	0	0	No	No	No
Ambulation loss (number of patients)	4	0	1	0	0	0	No	No	No
Cranial nerve involvement (number of patients)	Diaphragm weakness (5), vocal cord paralysis (5), facial palsy (2)	Facial palsy (1)	Tongue weakness and atrophy (1)	No	Vocal cord paralysis (1)	No	No	No	No
CMTESv2 (median/range)	22/between 21 and 24	19/between 13 and 21	14.5/between 10 and 16	10/7 and 13	15	6 and 9	12	16	NA
Median motor NCV (median/range)	12.0 (unobtainable in 4 patients)	3.8/between 3.0 and 9.4	12.9/11.2 and 15.0	9.0/between 6.2 and 25.0	16.0 and 26.0	28.0 and 42.0	27.4	19.0	Unresponsive
Other clinical features	NA	Severe sensory ataxia	NA	NA	NA	NA	NA	Hypogonadotropic hypogonadism	NA
Variant types (families)	TV (3) Missense (1)	TV (2)	TV (2)	TV (2)	TV (2)	Missense (1) TV (1) CNV (1)	TV	Missense	Missense
Zygosity (families)	Homozygous (4)	Homozygous (4)	Homozygous (4)	Homozygous (2)	Homozygous (2)	Homozygous (1) Compound heterozygous (1)	Homozygous	Heterozygous	Heterozygous

Abbreviations: CMT, Charcot‐Marie‐Tooth; CMTESv2, Charcot‐Marie‐Tooth examination score Version 2; DMM, Delayed motor milestones; NA, Not applicable; NCV, Nerve conduction velocity; PTV, Truncating variant.

In the CMT2 group, eight families carrying variants in five genes (*TRPV4*, *KIF1B*, *HSPB1*, *NEFH*, *LRSAM1*) were identified. Among them, four families had two different heterozygous *TRPV4* variants (p.[Arg232Cys], p.[Arg315Trp]) (Table 3).

**TABLE 3:** Clinical and genetic features of the patients with autosomal dominant axonal subtypes of Charcot–Marie‐Tooth 2 (CMT2) and intermediate Charcot–Marie‐Tooth (CMTi) subtypes.

Causative gene	*TRPV4*	*INF2*	*KIF1B*	*HSPB1*	*LRSAM1*	*NEFL*	*NEFH*	*ATP1A1*
Number of patients/families	4/4	2/2	1/1	1/1	1/1	1/1	1/1	1/1
Age at onset (years median/range)	12/between 5 and 25	8/7 and 9	6	13	7	1	7	11
Age at examination (years median/range)	25.5/between 25 and 30	14/13 and 15	25	37	41	19	20	21
Sex (female/male)	1/3	2/0	Male	Female	Female	Female	Female	Male
Consanguinity (number of patients)	0	0	Yes	No	No	No	No	Yes
Family history (number of patients)	3	0	No	No	Yes	No	No	No
DMM (number of patients)	0	0	No	No	No	Yes	No	No
Pes cavus (number of patients)	4	2	Yes	Yes	Yes	Yes	Yes	Yes
Hammer toes (number of patients)	3	1	Yes	Yes	No	No	Yes	Yes
Scoliosis (number of patients)	3	0	No	No	No	No	No	Yes
Other foot deformities (number of patients)	0	0	No	No	No	No	No	No
Tremor (number of patients)	3	0	No	No	No	Yes	No	No
Ambulation loss (number of patients)	0	0	No	No	No	No	No	No
Cranial nerve involvement (number of patients)	Vocal cord paralysis (3)	Hearing loss (2)	0	No	No	No	No	No
CMTESv2 (median/range)	11/between 6 and 12	9 each	4	4	15	18	5	10
Median motor NCV (m/s, median/range)	52.5/between 38.0 and 65.0	30.1/ 13.2 and 47.0	51.0	56.5	52.0	34.0	60.0	37.8
Other clinical features	Pyramidal signs	Nephropathy	NA	Mild pyramidal features	NA	NA	NA	NA
Variant types (number of families)	Missense (2)	Missense (2)	TV	TV	TV	Missense	TV	Missense
Zygosity (number of families)	Heterozygous (4)	Heterozygous (2)	Heterozygous	Heterozygous	Heterozygous	Heterozygous	Heterozygous	Heterozygous

Abbreviations: CMTESv2, Charcot‐Marie‐Tooth examination score Version 2; DMM, Delayed motor milestones; ID, Intellectual disability; NA, Not applicable; NCV, Nerve conduction velocity; PTV, Truncating variant.

The genetic distribution of the AR‐CMT2 subgroup was heterogeneous when *GDAP1* and *MFN2*‐related patients were excluded (Table 4) The most frequent subtype was *HINT1*‐neuropathy, with patients showing signs of peripheral nerve hyperexcitability observed either in neurological examination or electrophysiological tests. Seven different variants were identified, including two novel ones (p.[Phe33Leufs*22] and p.[Ser61Profs*8]). The recurrent p.(Arg37Pro) variant was found in three families. *SORD*‐neuropathy was identified in two probands. Both patients had the common homozygous p.(Ala253Glnfs*27) variant. In contrast, five more families with the same variant were identified in our dHMN cohort, making SORD‐neuropathy a frequent cause among dHMN/AR‐CMT2 patients. Two patients had the same homozygous p.(Lys177Asnfs*15) variant in the *MME* gene and disease onset was after the 4th decade in both patients similar to previously reported cases [11]. The rest of the AR‐CMT2 cohort includes singletons with variants in six different genes (Table 4 and Table S1).

**TABLE 4:** Clinical and genetic features of the patients with autosomal recessive axonal subtypes of Charcot–Marie‐Tooth 2 (AR‐CMT2).

Causative gene	HINT1	MME	SORD	MCM3AP	SACS	MPV17	MTRFR	IGHMBP2	SLC12A6
Patients/families	10/6	2/2	2/2	2/1	1/1	1/1	1/1	1/1	1/1
Age at onset (years median/range)	12/between 5 and 25	44.5/39 and 50	15 each	2/ 1 and 3	11	13	4	2	2
Age at examination (years median/range)	33.5/between 18 and 52	58.5/52 and 65	31.5/26 and 37	14/13 and 15	37	19	14	13	7
Sex (female/male)	6/4	0/2	0/2	0/2	0/1	0/1	1/0	1/0	1/0
Parental consanguinity (number of patients)	10	1	0	2	1	1	1	1	1
Family history (number of patients)	7	2	0	2	1	0	0	0	0
DMM (number of patients)	0	0	0	2	0	0	0	1	1
Pes cavus (number of patients)	5	2	2	2	1	1	1	0	0
Hammer toes (number of patients)	3	0	2	0	1	0	1	0	0
Scoliosis (number of patients)	0	0	0	0	0	0	0	0	0
Other foot deformities (number of patients)	0	0	0	0	0	0	0	0	1
Tremor (number of patients)	1	1	0	0	0	0	0	0	0
Ambulation loss (number of patients)	0	0	0	1	0	0	0	0	0
Cranial nerve involvement (number of patients)	Ophthalmoparesis (1), facial palsy (1)	0	0	0	0	0	0	0	0
CMTESv2 (median/range)	8.5/between 6 and 14	9 each	6.5/6 and 7	16.5/16 and 17	10	11	5	NA	NA
Median motor NCV (median/range)	47.5/between 42.0 and 55.0	41.9/ 39.0 and 44.8	39.5/34.0 and 45.0	23.0 and unobtainable	30.0	58.0	51.0	NA	NA
Other clinical features	Neuromyotonia, myokymia, muscle hypertrophy, intellectual disability, epilepsy, hyperhydrosis	NA	NA	NA	Mild pyramidal signs	Intellectual disability	Mild pyramidal signs	NA	NA
Variant types (number of families)	TV (2) Missense (2)	TV (1)	TV (1)	Missense (1)	TV	Missense	TV	Missense	TV
Zygosity (number of families)	Homozygous (6)	Homozygous (2)	Homozygous (2)	Homozygous (1)	Homozygous	Homozygous	Homozygous	Homozygous	Homozygous

Abbreviations: CCMTESv2, Charcot‐Marie‐Tooth examination score Version 2; DMM, Delayed motor milestones; NA, Not applicable; NCV, Nerve conduction velocity; TV, Truncating variant.

We identified two families with dominant intermediate CMT caused by the heterozygous missense variants in *INF2* (CMT‐DIE). Both had sensorineural hearing loss, one had a history of kidney transplant, and the other had microalbuminuria (Table 3).

We further detected pathogenic or likely pathogenic variants in genes unusual for a predominantly CMT phenotype including, *SPG7*, *FXN*, and *ATM*. The clinical and genetic features of these patients are summarized in Table 5.

**TABLE 5:** Clinical and genetic features of the patients with variants in genes that are unlikely to cause Charcot–Marie–Tooth disease.

Causative gene	*SPG7* ^a^	*SPG7*	*FXN* ^b^	*ATM*
Age at onset	18 months	25 years	3 years	25 years
Age at examination	26 years	28 years	16 years	35 years
Sex	Female	Female	Male	Females
Presenting symptom	Delayed walking	Numbness in feet	Delayed walking	Distal weakness in lower limbs
Parental consanguinity	2nd cousins	Absent	2nd cousins	Parents born in the same village
Family history	No	No	Yes	No
DMM	Yes	No	Yes	No
Pes cavus	Yes	No	No	No
Hammer toes	Yes	No	No	No
Scoliosis	No	No	Yes	No
Other foot deformities	No	No	Pes planus	No
Tremor	No	No	Yes	No
Pyramidal signs	Increased patella reflex	No	Increased patella reflex	No
Ambulation	Walks Independently	Walks Independently	Wheelchair‐bound	Walks Independently
Cranial nerve involvement	No	No	Optic atrophy	No
CMTESv2	9	Data not available	14.5/between 10 and 16	11
Median motor NCV	27.0 m/s	Data not available	46.9 m/s	51.1 m/s
Other clinical features	Bilateral cataracts	NA	Intellectual disability, mild cerebellar dysarthria, nystagmus, saccadic pursuit, seizures	NA
HGVS Transcript	NM_003119.4	NM_003119.4	NM_000144	NM_000051.4
Zygosity	Homozygous	Compound heterozygous	Homozygous	Compound heterozygous
Variant/DNA position	c.454A > G	Variant 1: c.1048C > A Variant 2: c.1553‐2_1553‐1del	c.493C > T	Variant 1: c.2377‐1G > A Variant 2: c.3576G > A
Variant/protein position	p.Met152Val	Variant 1: p.Pro350Thr Variant 2: ‐	p.Arg165Cys	Variant 1: ‐ Variant 2: p.Lys1192=

Abbreviations: CMTESv2, Charcot‐Marie‐Tooth examination score Version 2; DMM, Delayed motor milestones; HGVS, The Human Genome Variation Society; NA, Not applicable; NCV, Nerve conduction velocity.

^a^ Previously reported in Reference [11].

^b^ Previously reported in Reference [48].

## DISCUSSION

Herein, we described the clinical and genetic findings in CMT patients from a referral center in Türkiye by including the largest number of genetically diagnosed cases to date from the country. Previous studies from Türkiye were either performed on relatively smaller populations, including ours, that shared some patients from the current study or provided only an overview regarding the distribution [11, 13, 14, 15].

Overall, CMT1 was the most common subtype. It was unexpectedly followed by CMT4. This distribution shows a distinction when compared to other studies, where either CMTi or CMT2 is the second most common subtype [9, 16, 17]. Furthermore, *SH3TC2* and *GDAP1* variants were the second and third leading causes of demyelinating CMT, respectively. Interestingly, they were more common than *MPZ* variants, unlike in other populations [9, 12, 14, 17, 18, 19]. Four common CMT subtypes (*PMP22* duplication, *GJB1*, *MPZ*, and *MFN2*) accounted for 67% of our cohort compared to 85%–92% from previous reports [9, 16]. We assume this difference is caused by, first, the high frequency of complicated cases referred from different regions. Secondly, the relatively high rate of consanguinity in our country probably increases the frequency of rare autosomal recessive subtypes. Furthermore, the admixed but inbred characteristics of the population in Türkiye have given rise to an increased frequency of founder variants in genes described in various ethnic origins, such as *GDAP1*, *HINT1*, *SH3TC2*, and *NDRG1* [20, 21, 22, 23].

Twenty‐nine probands with *PMP22* duplication (24%) had a negative family history, which is higher than expected de novo mutation rate for *PMP22* duplication [24]. This can also be explained by the high rate of referral cases with a negative family history for further investigations to rule out acquired neuropathy causes.

CMTX1 was the only X‐linked subtype in our cohort. Disease onset was in the first or second decade in males except for two. As expected, due to random X‐inactivation, females had a later disease onset with lower CMTESv2 scores. In contrast, we did not observe a difference in median motor NCV between females and males, unlike previous studies [25, 26]. Interestingly, three patients (two males and one female) harboring the novel p.(Ile127Leu) variant had a later disease onset. CNS features were another intriguing feature in CMTX1. We previously reported our case with p.(Tyr211His) variant and further identified one patient with intellectual disability (p.[Arg230Cis]) and one with pyramidal signs (p.[Lys260Glufs*16]), which may be associated with the disease [27].

Patients with both monoallelic and biallelic variants in *MFN2* mainly presented in early childhood. Accordingly, 21 patients, including two with novel variants (heterozygous p.[Val222Ala] and homozygous p.[Val91Leu]), had pathogenic variants located in the Dynamin‐GTPase domain, which was previously associated with early disease onset. Unusual findings in patients with *MFN2* variants include pyramidal signs and cranial nerve palsies, as shown in previous studies [28]. Moreover, multiple symmetric lipomatosis was observed in two patients, which has only been rarely described in families harboring the p.(Arg707Trp) variant in at least one allele, similar to ours. Currently, the molecular mechanism underlying the association between this variant and the phenotype is largely unknown [29].

As expected, the striking clinical feature in patients with *SH3TC2* variants was the high frequency of kyphoscoliosis, observed in 77% of the patients. Interestingly, one patient had a median motor NCV > 38 m/s (40 m/s), which was rarely observed in these patients [30]. The disease severity in patients with other CMT4 subtypes was striking regardless of the pathogenic variant in terms of early ambulation loss and age of onset, high CMTESv2 scores, and frequent cranial nerve involvement. Patients with causative variants in the *GDAP1* gene may have an autosomal dominant or recessive inheritance as well as the presentation of either axonal or demyelinating nature [31]. All *GDAP1*‐associated CMT patients carried biallelic variants in our cohort. On the contrary, the presentation of neuropathy was heterogeneous in this group. Similarly, median motor NCV of patients with *MPZ*‐related CMT may fall into demyelinating, axonal, or intermediate range [5]. In fact, six patients had demyelinating, and two had intermediate CMT in patients with MPZ variants. Interestingly, median motor NCVs in two adult‐onset patients (22 and 53 years) were 15 and 17 m/s, unlike previous reports suggesting the association of later disease onset with axonal subtypes [32]. Furthermore, one patient showed conduction blocks mimicking CIDP, as described previously [26]. Other significant clinical clues in demyelinating CMT subtypes included vocal cord paralysis in *GDAP1*, ophtalmoparesis and tongue fasciculations in *FGD4*, severe sensory ataxia in *PRX*, respiratory involvement in *MTMR2* and *SBF2*, similar to previous reports [31, 33, 34, 35]. Indeed, one patient with a homozygous p.(Leu448Pro) variant in the *MTMR2* gene died at 18 years of age with complications due to respiratory muscle weakness. Intriguingly, one patient with a heterozygous *POLR3B* variant was diagnosed with hypogonadotropic hypogonadism, which was described in 4H leukodystrophy (OMIM#614381) patients with biallelic variants in the same gene.

The causative gene distribution in the CMT2 and AR‐CMT2 subgroups was highly heterogeneous. *HINT1* neuropathy was a common subtype in our cohort, especially when cases presenting with dHMN were considered. All patients with *HINT1* variants had signs of peripheral nerve hyperexcitability in neurological examination or electromyography. Interestingly, four of these patients had a history of febrile seizures, two had intellectual disability, and one had speech disturbances. CNS symptoms, such as psychiatric features and intellectual disability, were described previously in *HINT1* patients [36]. *SORD*‐neuropathy was another common subtype with a spectrum ranging from AR‐CMT2 to dHMN. These patients had a typical presentation with an onset at the 2nd decade with distal lower limb weakness. Clinical clues to pinpoint the causative gene from the heterogeneous CMT2 and AR‐CMT2 group included pyramidal signs in *SACS*, *MTRFR*, and *HSPB1*, vocal cord paralysis in *TRPV4*, intellectual disability in *MCM3AP*, late disease onset in *MME*, in accordance with previous reports [37, 38, 39, 40]. Mild intellectual disability was also observed in a patient carrying a pathogenic *MPV17* variant, which was described in patients with severe mitochondrial DNA depletion syndrome 6 [41]. We identified a novel heterozygous p.(Gln703*) variant in *KIF1B* in a patient with CMT2, which was classified as likely pathogenic according to the ACMG criteria. The patient's sequencing data was negative for other variants in CMT‐related genes. Although *KIF1B* was the first identified gene to cause CMT2, only a few pedigrees with *KIF1B* associations have been reported, and its pathogenicity remains controversial [42, 43, 44, 45]. On the contrary, a heterozygous missense variant in *KIF1B* was proposed to cause neuropathy by impairing insulin‐like growth factor 1 receptor (IGF1R) in a recent study [46].

The main phenotypic clue in patients with causative *INF2* variants was the history of kidney disease in various degrees ranging from asymptomatic proteinuria to renal failure requiring transplantation. Therefore, screening renal functions are essential for diagnosis and monitoring [47].

Various other genes can present with isolated CMT phenotype as well as with neuropathy as a part of a complex disease spectrum. Indeed, we identified four families with neuropathy as the predominant or sole clinical feature, harboring variants in genes causing complex neurological syndromes, such as *SPG7*, *FXN*, and *ATM* [48, 49, 50].

Disease severity was calculated with CMTESv2 in our study. Patients with autosomal recessive subtypes had a higher CMTESv2 when compared to dominant or X‐linked CMT subtypes.

Notably, our study also covers a period when high‐throughput sequencing techniques were not widely accessible. Therefore, several patients remained undiagnosed after sequential screening of common genes and did not undergo subsequent NGS tests. This may cause a negligible underrepresentation of rare CMT causative genes in our cohort.

Our findings increase the understanding of the genetic distribution of different CMT subtypes in a highly heterogeneous population, which is essential to creating population‐specific diagnostic workflows. This is particularly critical for Türkiye, as it is located at the crossroads between Europe and Asia, and the ethnic background is highly diverse. Furthermore, enriching the literature for clinical signs and disease severity will guide clinicians to pinpoint diagnostic clues.

## AUTHOR CONTRIBUTIONS

**Arman Cakar:** Methodology; data curation; writing – original draft; formal analysis; conceptualization; investigation; validation; resources; funding acquisition. **Ayse Candayan:** Investigation; writing – review and editing; formal analysis. **Gulandam Bagırova:** Investigation; formal analysis; data curation. **Zehra Oya Uyguner:** Writing – review and editing; investigation; formal analysis; data curation. **Serdar Ceylaner:** Data curation; investigation; formal analysis. **Hacer Durmus:** Investigation; data curation; writing – review and editing. **Esra Battaloglu:** Supervision; data curation; investigation; methodology; writing – review and editing; funding acquisition. **Yesim Parman:** Writing – review and editing; conceptualization; methodology; data curation; investigation; validation; supervision; funding acquisition.

## FUNDING INFORMATION

This study is partly funded by the Scientific and Technological Research Council of Turkey (TUBİTAK) with project No. 215S883 (EB and YP) and project No. 319S064 (Under the frame of EJP RD, the European Joint Programme on Rare Diseases—ENISNIP, ArmanC, GB, ZOU, HD, YP). The ENISNIP project has received further funding from the European Union's Horizon 2020 research and innovation programme under the EJP RD COFUND‐EJP N° 825575. Furthermore, EB and AyseC were supported by Bogazici University BAP small project grants #8341, #14784, and #17304.

## CONFLICT OF INTEREST STATEMENT

None of the authors have a conflict of interest to disclose.

## ETHICS STATEMENT

The study was approved by the Istanbul Medical Faculty Clinical Research Ethics Committee (Approval number: 2019/770) and complies with the agreements of the Declaration of Helsinki. Patients provided written informed consent before the procedures.

## Supporting information


**Table S1.** Genetic features of the identified variants in our cohort.

## Data Availability

The data that support the findings of this study are available from the corresponding author upon reasonable request.
